# *TP53* mutant *MDM2*-amplified cell lines selected for resistance to MDM2-p53 binding antagonists retain sensitivity to ionizing radiation

**DOI:** 10.18632/oncotarget.10073

**Published:** 2016-06-15

**Authors:** Catherine J. Drummond, Arman Esfandiari, Junfeng Liu, Xiaohong Lu, Claire Hutton, Jennifer Jackson, Karim Bennaceur, Qing Xu, Aditya Rao Makimanejavali, Fabio Del Bello, Alessandro Piergentili, David R. Newell, Ian R. Hardcastle, Roger J. Griffin, John Lunec

**Affiliations:** ^1^ Newcastle Cancer Centre, Northern Institute for Cancer Research, Medical School, Newcastle University, Framlington Place, Newcastle upon Tyne, United Kingdom; ^2^ Medicinal Chemistry Unit, School of Pharmacy, University of Camerino, Camerino, Italy

**Keywords:** MDM2 inhibitor, Nutlin-3, MI-63, resistance, MDM2-amplification

## Abstract

Non-genotoxic reactivation of the p53 pathway by MDM2-p53 binding antagonists is an attractive treatment strategy for wild-type *TP53* cancers. To determine how resistance to MDM2/p53 binding antagonists might develop, SJSA-1 and NGP cells were exposed to growth inhibitory concentrations of chemically distinct MDM2 inhibitors, Nutlin-3 and MI-63, and clonal resistant cell lines generated. The p53 mediated responses of parental and resistant cell lines were compared. In contrast to the parental cell lines, p53 activation by Nutlin-3, MI-63 or ionizing radiation was not observed in either the SJSA-1 or the NGP derived cell lines. An identical *TP53* mutation was subsequently identified in both of the SJSA-1 resistant lines, whilst one out of three identified mutations was common to both NGP derived lines. Mutation specific PCR revealed these mutations were present in parental SJSA-1 and NGP cell populations at a low frequency. Despite cross-resistance to a broad panel of MDM2/p53 binding antagonists, these *MDM2*-amplified and *TP53* mutant cell lines remained sensitive to ionizing radiation (IR). These results indicate that MDM2/p53 binding antagonists will select for p53 mutations present in tumours at a low frequency at diagnosis, leading to resistance, but such tumours may nevertheless remain responsive to alternative therapies, including IR.

## INTRODUCTION

The p53 tumour suppressor protein, encoded by the *TP53* gene, is post-translationally activated in response to a diverse range of cellular stresses and can lead to cell cycle arrest and apoptosis through both transcription dependent and independent mechanisms [1]. This process is tightly regulated by an autoregulatory feedback loop involving a direct protein-protein binding interaction between p53 and the product of the *MDM2* oncogene, which is also transcriptionally driven by p53. Once bound to p53, MDM2 inhibits p53 dependent transcription and also ubiquitinates the p53 protein to target it for nuclear export and proteasomal degradation. The importance of the p53 pathway in determining the appropriate response to such stresses is reflected by the high frequency with which p53 pathway abnormalities are observed in adult sporadic malignancies. In the approximately 50% of tumours that have a wild-type *TP53* gene upon diagnosis, other aberrations in the regulatory networks which control p53 activation are often observed [2–4] including amplification of the *MDM2* oncogene. Reactivation of wild-type p53 by small selective antagonists of the MDM2/p53 binding interaction is an attractive treatment strategy in these tumours [5].

The cis-imidazoline Nutlin-3 and the spiro-oxindole MI-63 are two compounds that have been developed as MDM2/p53 binding antagonists and shown to activate wild-type p53 both *in vitro* and *in vivo* [6, 7]. Studies with these compounds have supported the concept that non-genotoxic p53 activation might represent an alternative to current genotoxic chemotherapy in malignancies expressing wild-type *TP53*. Nutlin-3 and MI-63 increase the expression of p53 transcriptional target genes, such as those encoding p21WAF1 and MDM2, and induce cell cycle arrest and apoptosis in a p53 dependent manner. Both classes of antagonists have also been demonstrated to have *in vivo* activity [6, 8]. The first of this class of compound, RG7112 (Roche) has recently completed phase I clinical trials [9], whilst others, such as the spirooxindoles and the isoindolinones, which are being developed in this laboratory [10], are in late stage pre-clinical development.

Resistance to chemotherapy is associated with poor clinical responses and may either be due to intrinsic properties of the tumour or arise during the course of treatment. During the pre-clinical development of a novel class of anti-cancer agents it is useful to anticipate the mechanisms by which tumours may develop resistance to these agents. Many chemotherapeutic regimes induce multi-drug resistance by increasing the expression of export pumps such as p-glycoprotein (P-gp) and multi-drug resistance protein (MRP-1) in tumours and consequently the sensitivity of these tumours to a diverse range of chemotherapeutic agents is reduced [11]. Alternatively, treatment may induce or select for changes in the target that lead to resistance.

Intrinsic properties of tumours which may determine their initial sensitivity to MDM2/p53 binding antagonists have been extensively investigated in cell culture models and, as predicted from their mechanism of action, have confirmed the importance of wild-type p53. MDMX levels have also been proposed to play a role in determining the intrinsic sensitivity of cell lines to MDM2/p53 binding antagonists. MDMX is critically involved in the negative regulation of p53 alongside MDM2 and high levels of MDMX expression have been reported to correlate with reduced responses to Nutlin-3 [12, 13]. However, this is likely to be cell line specific as other studies have not identified MDMX as a major determinant of sensitivity to MDM2-p53 binding antagonists [14–16]. Established cell culture models have been used to investigate the susceptibility of Nutlin-3 to multi-drug resistance and the overexpression of P-gp was found to have little overall effect on sensitivity to Nutlin-3 as a single agent [17]. However, Nutlin-3 was found to be a P-gp substrate, and in this way inhibit P-gp mediated efflux of other drugs [18].

Studies, including those described here, have started to address how resistance to this class of compounds might develop during the course of treatment. Repeat exposure to Nutlin-3 was recently reported to induce p53 mutations in a cell culture models [19, 20]. Nutlin-3 has also been reported to increase markers of genotoxicity such as ɤ-H2AX and ATM autophosphorylation [21]. The generation of p53 mutations by Nutlin-3 during the development of resistance observed in these studies may be a specific response to Nutlin-3 mediated DNA damage rather than a general response to non-genotoxic reactivation of p53 by MDM2/p53 binding antagonists.

In this study, we investigated the mechanism by which resistance to MDM2/p53 binding antagonists may develop in more detail, using two chemically distinct classes of inhibitors. Two *MDM2*-amplified cell lines have been selected for resistance to growth inhibitory concentrations of Nutlin-3 and MI-63, and following the selection of resistant clones the mechanism of resistance has been characterised, including the response of the selected cells to ionizing radiation.

## RESULTS

**Figure 1:**
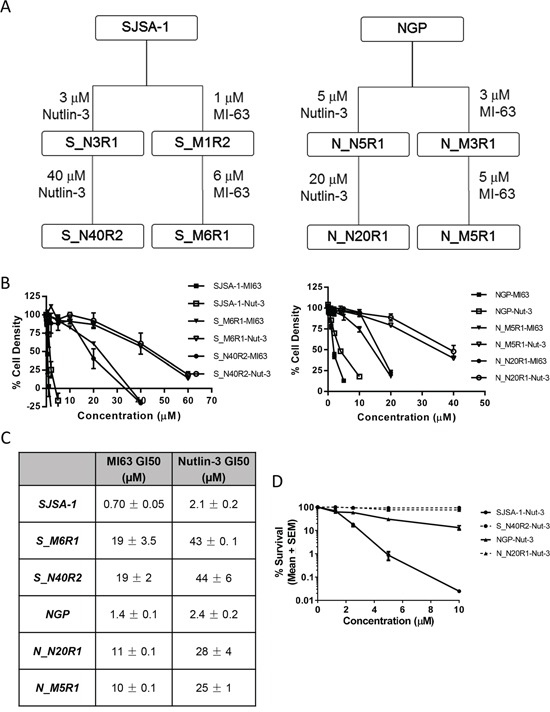
Selection of MDM2 inhibitor resistant clones SJSA-1 and NGP cell lines with resistance to MDM2/p53 binding antagonists were selected following 90 days exposure to either Nutlin-3 or MI-63 at the indicated concentrations **A.** GI_50_ doses of MI63 and Nutlin-3 for growth inhibition of parental and resistant SJSA-1 and NGP cell lines, together with the fold-differences in GI_50_ between resistant and parental cell lines **B.** Resistance was confirmed by GI_50_ (n≥3 ± SEM) determination following 72 hours of continuous exposure **C.** and by clonogenic survival following 48 hours of exposure **D.**

Cell lines with resistance to MDM2/p53 binding antagonists were generated by continuously exposing SJSA-1 osteosarcoma cells and NGP neuroblastoma cells to either Nutlin-3 or MI-63. MI-63 was used at a lower concentration than Nutlin-3 owing to its greater potency [7]. The SJSA-1 and NGP parental cell lines both have an amplified *MDM2* gene and express wildtype *TP53*. They are therefore a good model for the development of clinical resistance to this class of agents. S_M6R1 and S_N40R2 cells were developed from SJSA-1 cells following exposure to a final concentration of 6 μM MI-63 or 40 μM Nutlin-3 whilst N_M5R1 and N_N20R1 cells were developed from NGP cells following exposure to MI-63 or Nutlin-3 at final concentrations of 5 μM or 20 μM respectively (Figure 1A). The resistant sub-clones then underwent STR profiling to confirm that the selected daughter sub-clones were derived from their parental cell lines (Supplementary Figure S1).

### Cross-resistance to growth inhibition by different classes of MDM2-p53 binding antagonist

Resistance was confirmed by comparing the concentrations of MI-63 and Nutlin-3 required to inhibit the growth of these cell lines and parental cell lines by 50%. Following 72 hours continuous exposure to the antagonists, the cell lines generated were found to be resistant to both antagonists. In comparison to the parental SJSA-1 cells, S_M6R1 and S_N40R2 cells were found to be 21-fold resistant to Nutlin-3 and 27-fold resistant to MI-63. N_M5R1 and N_N20R1 cells were approximately 10-fold resistant to both antagonists (Figure 1B and 1C). Similar levels of resistance were observed by clonogenic cell survival assay (Figure 1D).

### MDMX expression

**Figure 2:**
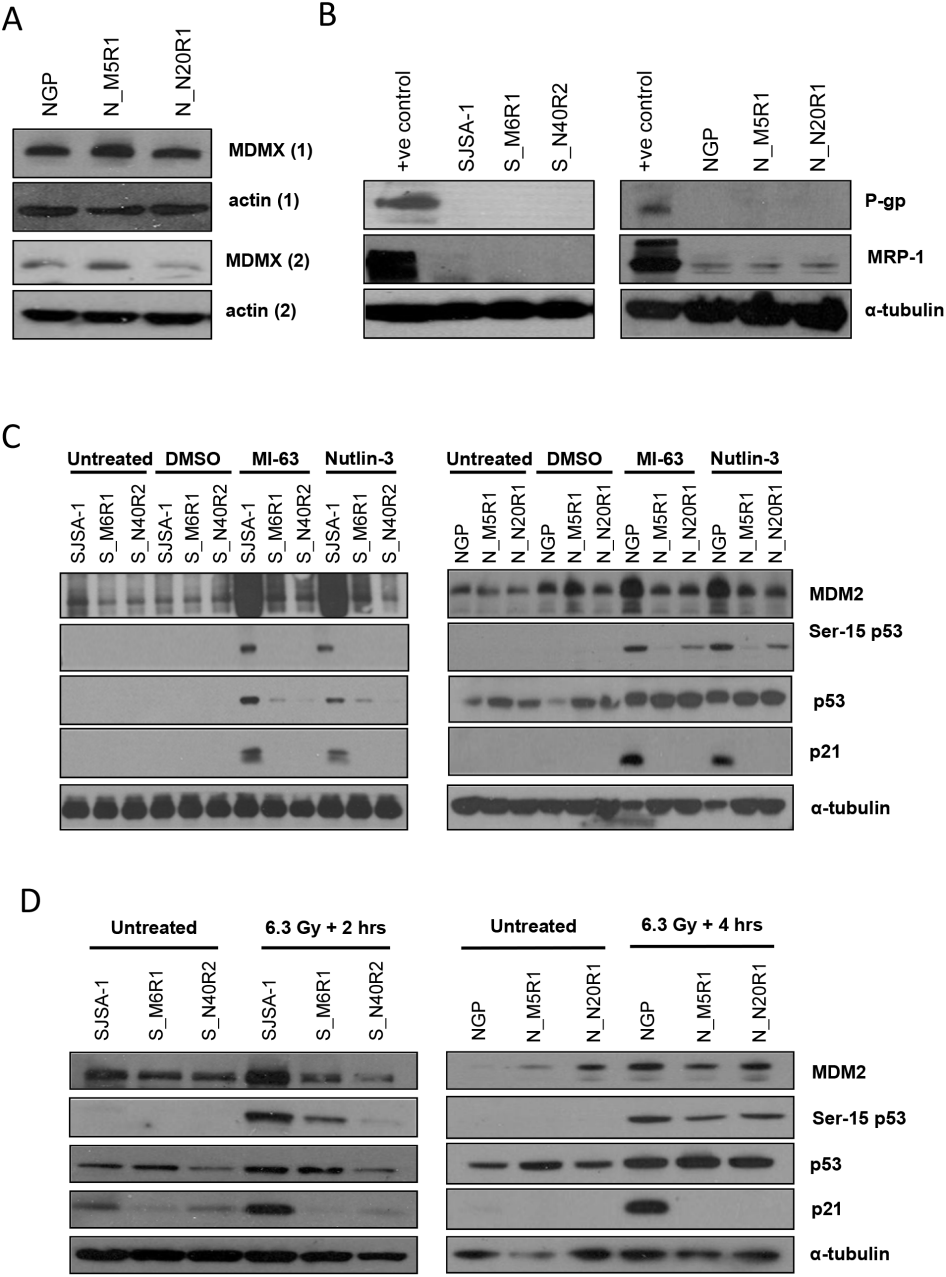
Investigating potential markers of resistance to MDM2-p53 binding antagonists Levels of MDMX protein (independent repeats 1 & 2) were determined by western blotting **A.** and levels of P-gp and MRP-1 protein by western blotting **B.** p53, ser-15 p53, MDM2 and p21^WAF1^ protein expression in parental and resistant SJSA-1 and NGP cell lines was compared by western blotting following 6 hours of exposure to Nutlin-3 (5 μM) or MI-63 (5 μM) **C.** and either 2 hours (SJSA-1) or 4 hours (NGP) following 6.3 Gy irradiation **D.** 1% DMSO was used as a vehicle only control.

Levels of MDMX protein were examined as a potential explanation for the observed resistance. In the NGP derived resistant lines MDMX expression was found to be comparable with the level observed in parental NGP cells (Figure 2A). MDMX expression was not detectable in the SJSA-1 cell line or its resistant derivatives (data not shown).

### P-glycoprotein (P-gp) and MRP-1 ABC transporter expression

As the development of resistance to chemotherapeutic agents is often associated with increased expression of the ABC transporters P-gp and MRP-1 [11] the level of P-gp and MRP-1 protein expression in the NGP and SJSA-1 derived cell lines with acquired resistance to MDM2/p53 binding antagonists was also determined. There was little evidence of P-gp protein expression in either the parental or resistance cell lines by western blotting and although low levels of MRP-1 protein were observed by western blotting in all of the NGP derived resistant cell lines, these levels were similar to those observed in parental NGP cells (Figure 2B). P-gp and MRP-1 expression was also examined in the NGP derived cell lines by flow cytometry and a small sub-population of resistant cells with higher levels of P-gp and MRP-1 than parental NGP cells was evident. However the median signal intensity was considerably less than the positive control MDCKII-MDR-1 (P-gp) and A549 (MRP) overexpressing cell lines (Supplementary Figure S2), consistent with the lack of significant difference seen on Western blot analysis.

### Nutlin-3 resistance and DNA damage

**Figure 3:**
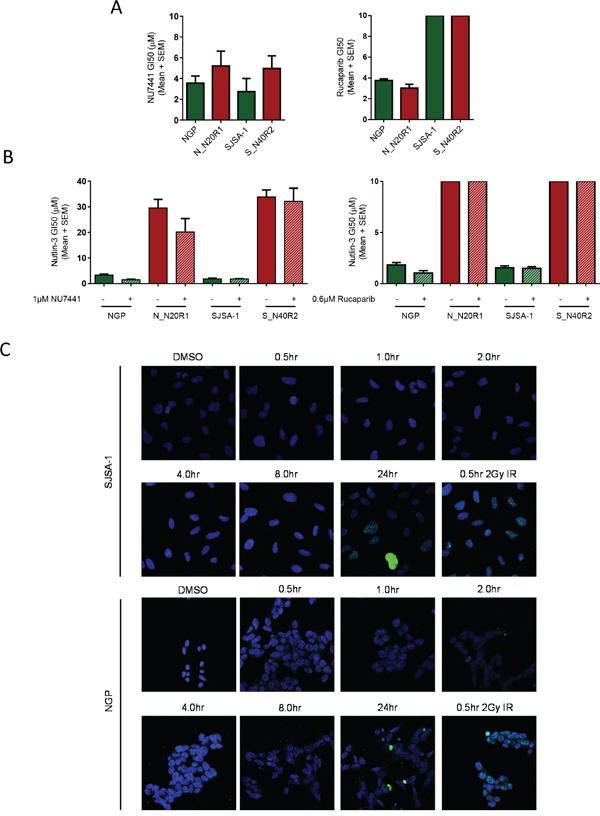
Inhibition of DNA-PK and PARP-1 do not influence the sensitivity of either the parental or their resistant counterparts to Nutlin-3 The growth inhibitory GI50 values for single agent NU7441/Rucaparib **A.**, or Nutlin-3 ± NU7441/Rucaparib **B.** in NGP and SJSA-1 cell lines and their most Nutlin-3 resistant counterparts (bars without an error indicate that the GI50 dose was >10). Confocal images of γ-H2AX immunofluorescent staining in SJSA-1 and NGP cell lines following treatment with 5μM Nutlin-3 (∼ 3x and 2x GI50 respectively) **C.**

In spite of the non-genotoxic mechanism of action attributed to Nutlin-3 by Vassilev *et al*, (2004), recent reports have suggested that Nutlin-3 may induce double strand breaks [21]. In which case, a potential mechanism of resistance may be enhanced DNA-repair activity or tolerance to genotoxic lesions [22]. To rule this out, we tested inhibitors of DNA-repair enzymes, DNA-PK and PARP-1, which have been shown to increase sensitivity to DNA damaging agents and could possibly reverse the resistant phenotype [23, 24]. There was no significant difference in sensitivity to single agent DNA-PK inhibitor (NU7441) or PARP-1 inhibitor (Rucaparib) between the parental and Nutlin-3 resistant daughter cell lines (Figure 3A). Furthermore, the presence or absence of NU7441 or Rucaparib did not impact Nutlin-3 mediated growth inhibition of the parental or the Nutlin-3 resistant daughter cell lines (Figure 3B).

### Kinetics of γ-H2AX staining after Nutlin-3 treatment

Immunofluorescence detection of γ-H2AX (Phospho-Ser139 histone H2AX) foci is an established marker of DNA double strand breaks. These foci can be detected within 30min of exposure to a relatively low dose of IR (<GI50 dose) (Figure 3C). However, γ-H2AX staining was not detected for ≤ 8 hours following treatment with up to three times the GI50 dose of Nutlin-3. Interestingly, focal and pan-nuclear γ-H2AX staining were observed 24 hours after treatment with Nutlin-3. However, this timing suggests that γ-H2AX staining following Nutlin-3 treatment only occurs much later and could be attributed to nucleolytic activity as a result of apoptosis and not through direct DNA damage by Nutlin-3. Data from another cell line (MCF-7) showed no significant dose-dependent increase in the integrated density of γ-H2AX immunofluorescent signals in response to 30 min treatment with Nutlin-3 (Supplementary Figure S3).

### Loss of p53 activation in resistant lines

The activation of p53 by MDM2/p53 binding antagonists is characterised by increased levels of p53 protein and transcriptional targets such as p21^WAF1^ and MDM2. To determine whether p53 is activated by Nutlin-3 and MI-63 in the resistant cell lines, western blotting was used to compare the levels of p53, p21^WAF1^ and MDM2 protein following exposure to the antagonists. The phosphorylation of p53 on serine 15 is considered to be a marker of genotoxic stress [25]. Although the original description of the Nutlins reported an absence of p53ser15 phosphorylation as evidence for non-genotoxicity [6], it is consistently observed in response to both Nutlin-3 and MI-63 [26].

Consistent with the activation of p53, the levels of total p53 protein, p21^WAF1^ and MDM2 increased in both parental SJSA-1 and NGP wild-type p53 cell lines. Phosphorylation of p53 on serine 15 was also detected on treatment with the MDM2 inhibitors in both parental cell lines (Figure 2C).

Interesting differences between the accumulation and phosphorylation of p53 protein in the SJSA-1 and NGP derived resistant cell lines were observed. In the SJSA-1 derived resistant lines, increases in p53 protein were only observed in S_M6R1 cells and were much lower than that observed in parental SJSA-1 cells. Nutlin-3 induced p53 protein stabilisation was notably attenuated in the S_M6R1 and S_N40R2 cells. No Phosphorylation of p53ser15 was observed in either S_M6R1 cells or S_N40R2 cells. In contrast, increased baseline levels of p53 protein were evident in both of the NGP derived resistant lines, but these did not increase markedly in response to treatment with Nutlin-3 or MI-63 in comparison to the increases observed with the parental NGP cells. Phosphorylated p53 was also observed in both Nutlin-3 and MI63 treated N_N20R1 cells and to a lesser extent in N_M5R1 cells, but not to the same extent as seen with the parental NGP cells. Despite these differences, Nutlin-3 and MI63 failed to functionally activate p53 in the resistant lines, since no increases in the expression of p53 target genes MDM2 and p21^WAF1^ were observed in either S_M6R1 and S_N40R2, or N_M5R1 and N_N20R1 cells in response to either antagonist (Figure 2C).

### Loss of p53 activation in response to genotoxic stress

Next, the phosphorylation of p53 on serine 15 and the levels of p53, MDM2 and p21^WAF1^ protein were compared following X-irradiation in the parental and resistant lines to determine whether p53 is activated by genotoxic stress. The levels of both total and phosphorylated p53 protein increased in response to 6.3Gy of X-irradiation in the parental cells lines, and was transcriptionally active, as shown by increases in the expression of p53 target genes MDM2 and p21^WAF1^ (Figure 2D). In both NGP derived lines and S_M6R1 cells p53 protein levels also increased in response to irradiation, but to a lesser extent than seen in the parental cell lines. Despite these increases, no changes in MDM2 and p21^WAF1^protein levels were observed in any of the resistant lines. In this regard, the response of the resistant lines to irradiation was similar to that of p53 deficient HCT116 cells (Supplementary Figure S4). Interestingly, phosphorylation of p53 on serine 15 was observed in response to X-irradiation in both N_M5R1 and N_N20R1 cells and in S_M6R1 cells following irradiation, and hence in this respect the response of these lines to the MDM2/p53 binding antagonists and to genotoxic stress differed. Together these results suggest that although p53 accumulation is observed, there is no evidence of p53-dependent transcriptional transactivation by either Nutlin-3 and MI-63 or ionizing radiation in any of the cell lines selected for resistance to MDM2/p53 binding antagonists.

### DNA sequence and TP53 FISH analysis

**Figure 4:**
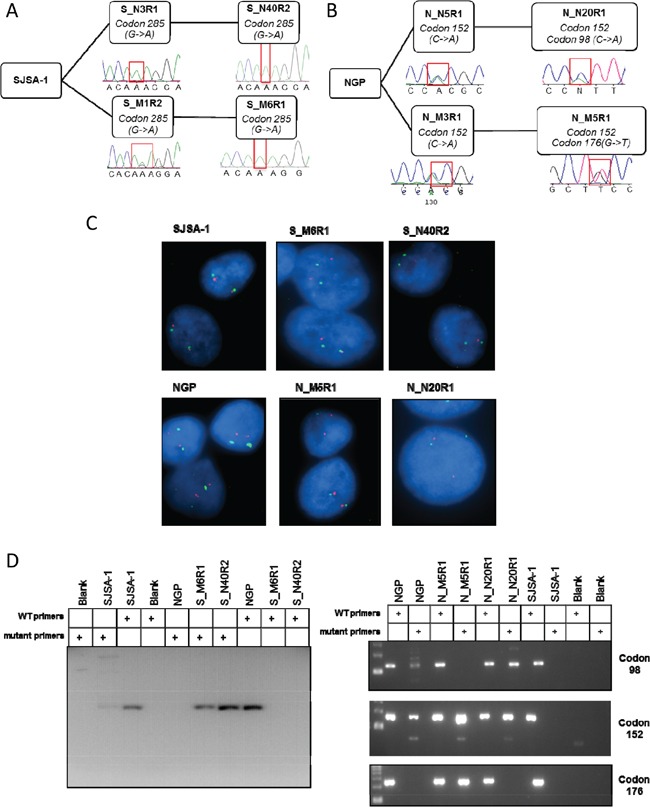
The MDM2 inhibitor resistant sub-clones are *TP53* mutant and detectable in the parental population Chromatograms showing *TP53* mutations in resistant cell lines at both stages of selection in SJSA-1 **A.** and NGP **B.** derived lines. Representative FISH images showing *TP53* copies (red) in parental and resistant cell lines together with centromeric chromosome 17 (green) signals using a dual probe against p53 and chromosome 17 centromeric DNA **C.** Mutation specific PCR detecting the presence of low frequency mutations in parental SJSA-1 (A) and NGP (B) cell cultures. Primers specific to the identified mutations in codons 285, 98, 152 and 176 were used as indicated, and the codon 285 mutation in parental SJSA-1 cells was visualized by inverting the image for clarity **D.**

Mutations in the DNA binding domain of p53 occur in ∼ 50% of tumours and have been reported in cells selected for resistance to Nutlin-3 [19, 20]. To determine whether p53 mutations are present in the SJSA-1 and NGP derived cell lines with resistance to both MI-63 and Nutlin-3 described here, exons 4 to 9 of p53, which encode the DNA binding domain, were sequenced. Parental SJSA-1 and NGP cells were found to have wild-type p53 as previously reported [27, 28]. Point missense mutations resulting in amino acid substitutions were detected in all of the resistant cell lines (Figure 4A and 4B). A homozygous G>A transition resulting in a Glu285Lys mutation was found in both the S_N40R2 and S_M6R1 resistant cell lines derived from SJSA-1, whilst a heterozygous C>A transversion resulting in a Pro152Thr mutation was detected in both of the NGP derived cell lines. The NGP derived lines were also found to have additional point mutations; A G>T transversion resulting in a Cys176Phe heterozygous mutation was detected in the N_M5R1 cells whilst a C>A transversion resulting in a Pro98His mutation was found in the N_N20R1 cells.

Loss of heterozygosity is commonly observed in conjunction with p53 mutations and would account for the homozygous mutation observed in the SJSA-1 derived cell lines. This was investigated further with fluorescent *in situ* hybridisation (FISH) (Figure 4C and Table 1). The parental SJSA-1 and NGP cell lines were confirmed to have two *TP53* alleles (94% and 95% respectively), as were the NGP-derived N_N20R1 (93%) and N_M5R1 (92%) cells. The loss of one *TP53* copy was evident in the S-N40R2 cell line, with 68% of cells containing a single copy of *TP53*, and a further 14% having lost both copies. Whilst the majority of S_M6R1 cells had two copies of *TP53* (77%), a single copy was observed in 20% of the population (Table 1). The predominant presence of two copies of *TP53* in the S_M6R1 cell line and the clear evidence of a homozygous mutation in the sequence analysis (Figure 4A) indicates uniparental disomy, in which a deletion of a normal *TP53* allele has been accompanied by a duplication of the mutated allele.

**Table 1:** Cell lines and their *TP53* genetic and cytogenetic status

Cell Line	Exon	Codon	Mutation	Amino Acid Substitution	17p status
***SJSA-1***	-	-	-	-	2R2G: *99/106 (94%)* 1R2G: *5/106 (4.7%)*
***S-M6R1***	Exon 8	285	GAG→AAG (homozygous)	Glu → Lys	2R2G: *102/133 (77%)* 1R2G: *17/133 (13 %)* 2R4G: *9/133 (6.8%)*
***S-N40R2***	Exon 8	285	GAG→AAG (homozygous)	Glu → Lys	2R2G: 6/100 (6%) 1R2G: 68/100 (68%) 1R3G: 6/100 (6%) 0R2G: 14/100 (14%)
***NGP***	-	-	-	-	2R2G: 97/102 (95%)
***N-N20R1***	Exon 4 Exon 5	98 152	CCT→CAT (heterozygous) CCG→ACG (heterozygous)	Pro→His Pro→Thr	2R2G: 102/110 (93%) 1R2G:7/110 (6.4%)
***N-M5R1***	Exon 5 Exon 5	152 176	CCG→ACG (heterozygous) TGC→TTC (heterozygous)	Pro→Thr Cys→Phe	2R2G:106/116 (92%) 1R2G:7/116 (6.4%)

Details of *TP53* mutations and chromosome 17p status established by FISH in cell lines selected for resistance to MDM2-p53 binding antagonists. R (Red), G (Green) refer to the number and percentage of 17p (*TP53*) and centromeric signals respectively.

*TP53* mutations occur in response to genotoxic stress. Despite being believed to activate p53 by a non-genotoxic mechanism, both Nutlin-3 and MI-63 were found to induce phosphorylation of p53 on serine 15, a known marker of genotoxic stress (Figure 2C). To establish whether DNA damage is implicated in the phosphorylation of p53 on serine 15 observed in response to Nutlin-3 and MI63, the dependence of this phosphorylation on the DNA damage dependent ATM and DNA-PK kinases was assessed.

The p53 ser15 phosphorylation occurred less rapidly in response to Nutlin-3 and MI-63 than in response to X-irradiation (Supplementary Figure S5A). The SJSA-1 cells were then pre-treated with the ATM specific inhibitor KU55933 [29] and either exposed to Nutlin-3 or MI-63, using X-irradiation as a positive control (Supplementary Figure S5B). As expected, KU55933 was observed to reduce p53 serine 15 phosphorylation following irradiation. However, serine 15 phosphorylation induced by the MDM2/p53 antagonists was not effected by KU55933. Specific inhibitors of the DNA damage activated kinase DNA-PK, KU-0060648 and NU7441 [30], were also found to have little effect on Nutlin-3 and MI-63 induced phosphorylation of p53 on serine-15 (Supplementary Figure S5B). Together, these results show that neither Nutlin-3 nor MI-63 are inducing DNA damage and therefore these agents themselves are not responsible for the p53 mutations observed in the selected resistant cell lines.

Next, mutation specific PCR was used to investigate whether Nutlin-3 and MI63 treatment had selected for pre-existing mutations which might be present at a low level in the parental cell populations. Mutation specific PCR is more sensitive than sequencing by PCR based Sanger dideoxy chain termination and has been previously shown in this laboratory to detect mutations present in 0.05% of the population [3]. Consistent with the sequencing results, there was no evidence of wild-type p53 in either the S_M6R1 or the S_N40R2 cell lines and a PCR product was obtained with primers specific to the codon 285 mutation in both of these cell lines (Figure 4D). Similarly the presence of mutations in codons 98, 152 and 176 were confirmed in N_M5R1 and N_N20R1 cells as predicted by sequencing (Figure 4D). Low levels of PCR product was detected in SJSA-1 parental cells using the mutant codon 285 primers and in NGP parental cells using the mutant 152 primers and 98 primers.

### Downstream cellular effects on growth and apoptosis

Next, comparisons between changes in cell cycle distribution and the level of apoptosis observed in the parental cell lines and their resistant counterparts were made following exposure to the MDM2-p53 binding antagonists. Both p53-dependent cell cycle arrest and apoptosis are well characterised responses to MDM2/p53 binding antagonists that occur following the activation of p53 by these agents and result in growth inhibition and anti-tumour activity.

**Figure 5:**
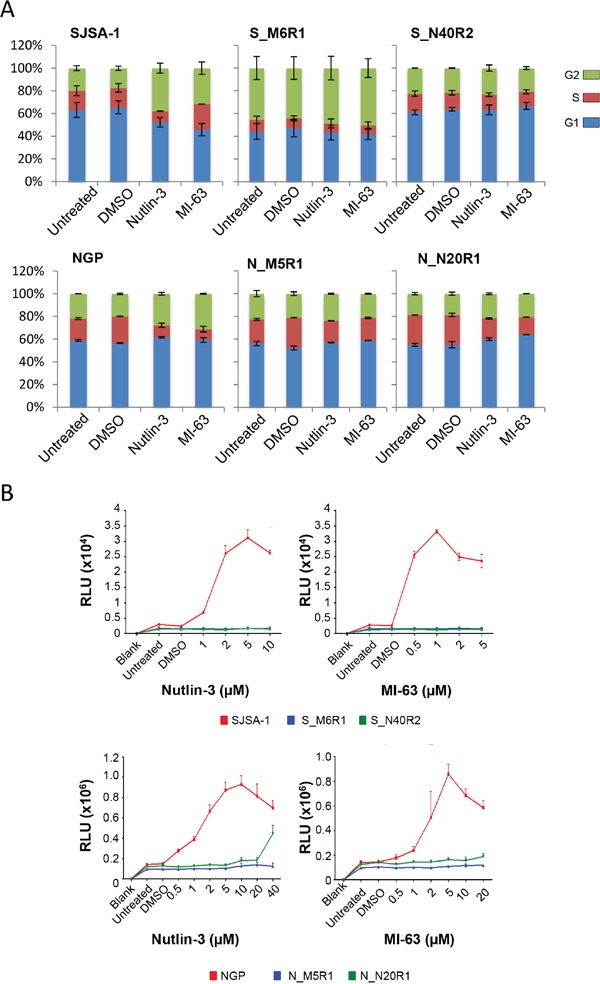
Downstream effects on cell cycle progression and apoptosis The effects of Nutlin-3 (5 μM) and MI-63 (5 μM) on cell cycle distribution in parental and resistant SJSA-1 and NGP cell lines determined by flow cytometry following 24 hours of exposure (n=2 ± SEM) **A.** Caspase 3/7 Glo assays showing the level of caspase 3 and 7 activation by Nutlin-3 and MI-63 in SJSA-1 and NGP parental and resistant cell lines following 48 hours of exposure **B.**

In response to 24 hours of exposure to the antagonists, SJSA-1 parental cells were observed to accumulate in G2/M phase. The S-phase fraction of MI-63 treated cells was similar to that observed in untreated cells and potentially reflects an S-phase arrested sub-population. The percentage of cells with sub-G1 DNA content increased in response to both antagonists (data not shown). In contrast, Nutlin-3 and MI-63 had little effect on the cell cycle distribution or the induction of apoptosis in the resistant S_M6R1 and S_N40R2 cell lines (Figure 5A).

Nutlin-3 and MI-63 were similarly found to induce cell cycle arrest in parental NGP cells, whilst having little effect on the NGP derived N_M5R1 and N_N20R1 cells. An increase in both G1 and G2/M phase cells and a corresponding decrease in S phase cells was observed in NGP cells in response to both MDM2-p53 binding antagonists, whereas smaller changes in cell cycle distribution were evident in the resistant lines (Figure 5A). Interestingly, with the SJSA-1 cells there is predominantly a G2 arrest, but with the NGP cells both G1 and G2 increases are seen.

Apoptosis was also induced by the MDM2-p53 binding antagonists in the parental cell lines. Following 48 hours of exposure, the caspase-3/7 activity increased in response to both of the antagonists in a dose-dependent manner (Figure 5B). In contrast, by comparison there was little evidence of the resistant lines undergoing apoptosis following 48 hours of exposure to MI-63 or Nutlin-3. Increases in caspase-3/7 activity were not observed in S_M6R1, S_N40R2 and N_M5R1 cells, whilst in N_N20R1 cells were only observed following exposure to 40 μM Nutlin-3, a high dose at which off-target p53-independent side effects start to be seen.

The inability of Nutlin-3 and MI-63 to induce either cell cycle arrest or cytotoxicity in the resistant cell lines is consistent with their inability to activate p53-dependent transcription in these cells.

### Downstream cellular responses to ionizing radiation

**Figure 6:**
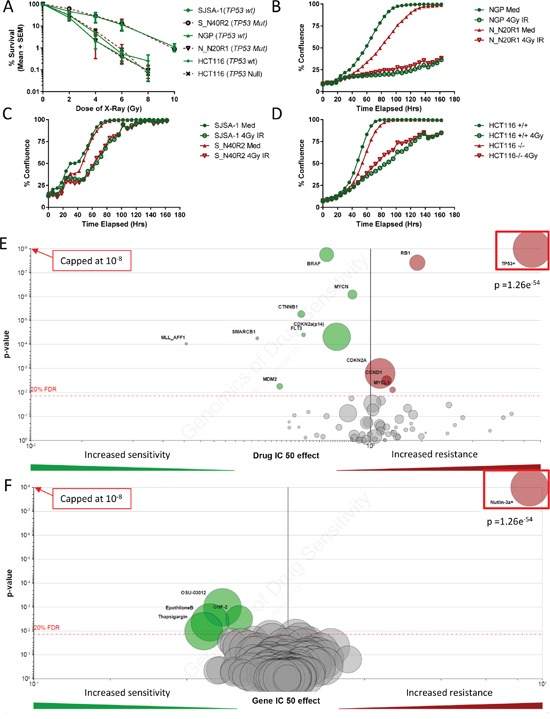
MDM2-p53 binding antagonist resistant daughter cells retain their sensitivity to ionizing radiation irrespective of their p53 status Clonogenic survival curves of MDM2 inhibitor sensitive parental and resistant daughter cell line pairs along with HCT116p53+/+ & −/− isogenic paired cell lines, in response to ionizing radiation **A.** Percentage confluence of the two otherwise isogenic MDM2 sensitive and resistant cell line pairs **B.** and **C.** and HCT116p53+/+ & −/− isogenic cell line pair **D.** over time using Incucyte; in response to 0 or 4Gy ionizing radiation. Volcano plots show the effect that common genetic alterations in cancer have on cell line sensitivity to Nutlin-3a **E.**, and the effect that *TP53* mutations have on sensitivity of a large panel of genotoxic and non-genotoxic anticancer compounds **F.** For further explanation of the volcano plot please see http://www.cancerrxgene.org.

To investigate the response of the cell lines with acquired resistance to MDM2/p53 binding antagonists to conventional therapy we assessed their response to DNA damage in the form of X-radiation. The results showed there was no difference between the parental and resistant cell lines in their clonogenic survival following X-irradiation. Similarly there was no difference in the clonogenic cell survival response to X-irradiation between the HCT116 p53 +/+ and p53 −/− isogenic cell line pair (Figure 6A). The response to X-irradiation of the sensitive and their MDM2 inhibitor resistant daughters was unaffected in short term growth inhibition assays (Figures 6B, 6C and 6D). This is consistent with the genomics of drug sensitivity data made available by the Wellcome Trust Sanger Institute (Figure 6E and 6F). These data show that the genetic status of *TP53* is the most significant determinant of growth inhibitory response to Nutlin-3a (The active enantiomer of Nutlin-3 racemic mixture) (Figure 6E). Conversely, *TP53* genetic status is not flagged up as a determinant of growth inhibitory response to any of the genotoxic agents used in their panel of compounds (e.g. Doxorubicin) (Figure 6F). These observations overall strongly support the notion that MDM2 inhibitors result in selection of *TP53* mutant clones and that these clones are still likely to remain sensitive to DNA damaging agents.

## DISCUSSION

MDM2/p53 binding antagonists have been demonstrated to have anti-tumour activity in *in vivo* xenograft models [6, 8], and their clinical potential for the treatment of p53 wild-type tumour types has been confirmed by initial results from phase I clinical trials that have focused on leukaemia and sarcoma, which are predominantly p53 wild-type at diagnosis and are *MDM2*-amplified or have high expression of MDM2 [9]. However, the development of resistance to these agents is likely to influence their effectiveness in the clinic and knowledge of them is required to inform follow-up strategies. In this study, the mechanism through which resistance to these antagonists might develop in cells with amplification and overexpression of *MDM2* has been investigated in detail with two chemically different binding antagonists, MI-63 and Nutlin-3.

*MDM2*-amplification and *TP53* mutation are widely and consistently reported as mutually exclusive, so that the selection for *TP53* mutation in the context of *MDM2* amplification represents a novel genotype that may occur in therapeutic situations, particularly with those cancers in which *MDM2*-amplification is prevalent, such as sarcomas, against which MDM2 inhibitors have demonstrated activity in clinical trials. Hence the need to investigate selected resistant cells with this unusual genotype. This has clear clinical relevance for what potential combination or follow on treatment options might be appropriate and provides a mechanistic rationale. This has not been previously investigated. Resistant cell lines were developed from two p53 wild-type, *MDM2*-amplified cell lines of different tumour types, with S_M6R1 and S_N40R2 cells originating from the SJSA-1 osteosarcoma cell line, and N_M5R1 and N_N20R1 cells from the NGP neuroblastoma cell line [27, 31]. Following selection these cell lines were found to be resistant to both MI-63 and Nutlin-3 as well as the isoindolinones, a chemically distinct class of MDM2/p53 binding antagonists being developed in this laboratory [32].

Slight increases in both P-gp and MRP-1 protein expression was detected in all of the NGP derived cell lines by flow cytometry. Nutlin-3 is a known substrate of P-gp [18] and whilst the susceptibility of MI-63 to transport mediated resistance is unknown, these results suggest that MI-63 is also a substrate of both P-gp and MRP-1. However, as the increases observed were an order of magnitude lower than those observed in the positive control MDCKII and A549 cells, increases in the expression of drug efflux pumps following long term exposure to Nutlin-3 or MI-63 are unlikely to account for their resistance to these agents.

There was no significant difference in sensitivity to the DNA repair enzyme inhibitors NU7441 and Rucaparib as single agents, between the parental cell lines and their resistant counterparts. This strongly suggests that there is no difference in DNA repair processes or elevated tolerance to DNA damage between the parental and resistant cell line pairs. Interestingly, NGP and N_N20R1 cells were found to be sensitive to single agent Rucaparib, which suggests that both the parental NGP cells and their resistant clones may have a low homologous recombination (HR) DNA repair capacity [33]. However, this reduced HR capacity in the NGP cells does not make them more sensitive to MDM2 inhibitors than the SJSA-1 cells.

Concomitant treatment with Nutlin-3 ± NU7741/Rucaparib did not influence the growth inhibitory sensitivity to Nutlin-3 of neither NGP and N_N20R1 nor SJSA-1 and S_N40R2 cell line pairs. These data, along with the absence of γ-H2AX staining for up to 8 hours of treatment with Nutlin-3, are consistent with the non-genotoxic mechanism of action of Nutlin-3 (Figure 3).

In comparison to the increases in MDM2 and p21^WAF1^ expression observed in the parental lines, there was no evidence of p53 dependent transactivation being induced by either MDM2-p53 binding antagonists or ionizing radiation in any of the resistant cell lines. Furthermore, the cell cycle arrest and apoptosis observed in response to Nutlin-3 and MI-63 in both parental cell lines was not observed in either the SJSA-1 derived lines or the NGP derived cell lines. Mutations within the DNA binding domain of p53 were subsequently identified accounting for the demonstrated abrogation of p53 function. *TP53* mutations were found in codon 285 in the SJSA-1 resistant lines and codons 98, 152 and 176 in the NGP derived lines. Whilst these residues are not one of the 6 residues considered to be ‘hotspots’ [34], mutations in codons 152, 176 and 285 occur frequently in multiple tumour types. Codon 98 mutations have been documented less frequently [35] although, P98H has been reported by the IARC TP53 mutation database to result in inactive p53.

Intra-tumour heterogeneity for p53 status has been demonstrated in a number of different tumour types, and the identical nature of the mutation identified in both SJSA-1 derived lines, and of one of the three p53 mutations found in NGP derived lines demonstrated here, suggests that these were present in the original populations at low frequencies and were selected for during the course of treatment. Whilst these results are in contrast with previous studies which found Nutlin-3 to be inducing p53 mutations [19, 20], the generation of identical mutations by two chemically distinct binding antagonists on multiple occasions is unlikely and as a class of agents, MDM2/p53 binding antagonists are considered to be non-genotoxic reactivators of wild-type p53 [6]. The phosphorylation of p53 ser15 by Nutlin-3 and MI-63 observed here was independent of both ATM and DNA-PK, consistent with these antagonists acting through DNA damage independent mechanisms. Furthermore, the results presented here support the findings of Jones *et al.*, who identified identical p53 mutations in H929 cells selected independently for resistance against Nutlin-3 and MI-63 [36]. All but one of the mutant alleles was detected in the parental cell populations by mutation-specific PCR, indicating that the binding antagonists had selected for existing p53 mutations present in the parental populations. However, we cannot rule out that the codon 176 mutation in the N_M5R1 cell line was present below the level of detection by mutation specific PCR in the parental cell population, or was a *de novo* mutation occurring during the selection procedure.

The demonstration that long term exposure to low concentrations of MDM2-p53 antagonists appears to select for p53 mutations which might be present at low levels in parental populations is of direct therapeutic relevance. The selection of p53 mutant populations occurred in response to two chemically distinct classes of binding antagonists with two different cell lines and therefore identified a common mechanism by which resistance to this class of agent will develop. These mutations are likely to have occurred spontaneously in the parental populations, and then been selected for following p53 activation and the growth inhibition or apoptosis of cells expressing wild-type p53. Whilst NGP cells were derived from a patient who had received prior chemotherapy, SJSA-1 cells originated from a patient having had received no prior treatment and hence these mutations are unlikely to have originated during prior treatment. If the selection for *TP53* mutant sub-clones was reflected in a therapeutic setting, it would suggest that patients with resistance to MDM2/p53 binding antagonists would benefit from follow up treatment with agents acting through p53 independent mechanisms. In this respect, it is notable that the response of both the NGP and SJSA-1 resistant cell lines to ionizing radiation was similar to that of parental NGP and SJSA-1 cells. This is consistent with information on the Sanger Genomics of Drug Sensitivity database, which shows no significant difference in response to a wide range of DNA damaging agents for cell lines of wild-type compared with mutant p53 status, despite demonstrating a very marked difference in response to Nutlin-3 (Figure 6E and 6F). It is also consistent with p53 dependent apoptosis having a relatively modest role in cellular response to ionizing radiation, as reviewed by Gudkov *et al* [37].

*MDM2*-amplification and p53 mutations are mutually exclusive events and the otherwise isogenic paired cell lines generated here are among the first examples of this. The effect of *MDM2*-amplifications in a mutant p53 background is currently unknown and how this may affect the efficacy of current chemotherapeutic agents is an important consideration for treating patients following MDM2/p53 binding antagonist therapy. These results presented here suggest these patients will remain responsive to conventional therapy. Derived resistant cell lines remained responsive to X-irradiation and classical multi-drug resistance is unlikely to be a consideration for these patients, as P-gp and MRP-1 expression remained low during the development of resistance. The paired cell lines generated here provide the opportunity to investigate this in greater detail, as well as being a valuable resource for testing the cellular specificity of novel MDM2-p53 binding antagonists.

## MATERIALS AND METHODS

### Reagents

Nutlin-3 (Immunodiagnostic Systems Ltd) and MI-63 (synthesised by Siena Biotech S.p.A) were stored as stock solutions in DMSO at −20°C and diluted in culture medium immediately prior to use. KU55933 [29] and KU-0060648 [38] were provided by Kudos Pharmaceuticals, and NU7441 [30] was developed in this laboratory. Rucaparib (AG-014699) was kindly provided by Prof. Nicola J. Curtin.

### Cell culture and generation of resistant cell lines

Resistant cell lines were established by exposing NGP and SJSA-1 cells to Nutlin-3 or MI-63. Single cell derived colonies were isolated with cloning cylinders and the clonal population expanded in culture medium containing the MDM2/p53 antagonist refreshed weekly for 60 days. Stage 1 resistant clones were then further exposed to increased concentrations of Nutlin-3 or MI-63 for 30 days to generate stage 2 resistant clones (Figure 1A). NGP and SJSA-1 parental cell lines and resistant clones were sub-cultured weekly, and were not used beyond 30 passages post authentication by short tandem repeat (STR) profiling (LGC Standards).

### Cell growth inhibition assays

GI_50_ (50% growth inhibitory concentration) values were determined by the sulforhodamine B (SRB) staining method [39]. Cells were seeded in 96-well plates (SJSA-1; 2×10^3^ cells/well, NGP; 5×10^3^ cells/well) and allowed to attach for 24 hours before exposure to Nutlin-3 or MI-63 for 72 hours. Nutlin-3 and NU7441/Rucaparib were administered concomitantly. Cells were fixed in Trichloroacetic Acid (10% v/v), stained with SRB (0.4% w/v) and the absorbance measured at 570 nM using a SpectroMax 250 (Molecular Devices, Berkshire, UK) microwell plate scanner. GI_50_ values were calculated using GraphPad PRISM software (GraphPad Software, Inc., San Diego, CA, USA) and fold-resistance determined. For assessment of cell confluence and proliferation over time, cells were seeded 24 hours before treatment and confluence was measured by IncuCyte^®^ Zoom (Essen BioSciece) every 6 hours.

### Western blotting

Western blotting was performed as previously described [2] using primary antibodies raised against P-glycoprotein (1:100, sc-13131: Santa Cruz Biotech, CA, USA), MRP-1 (1:200, sc-18835, Santa Cruz Biotech), MDM2 (1:100, IF2; EMD Chemicals Inc., Gibbstown, NJ, USA), p53 (1:300, DO-7; Novocastra Laboratories, Newcastle, UK), serine 15 pp53 (1:1000, # 9284; Cell Signalling, Danvers, MA, USA) and p21^WAF1^ (1:100, EA10, EMD Chemicals Inc). α-Tubulin (DM1A; Sigma-Aldrich, Dorset, UK) was used as a marker of protein loading.

### Flow cytometry for P-gp and MRP-1

Harvested cell samples were pre-incubated with block solution (phosphate buffered saline (PBS) + 10% Foetal Bovine Serum) for 12-16 hours, washed twice with cold PBS and incubated for 30 minutes at 4°C with either FITC anti-P-gp (17F9) or isotype-matched FITC Mouse IgG2a control (BD Bioscience, Oxford, UK). Intracellular staining with FITC anti-MRP1 (QCRL-3) was carried out using the Intra-stain Fix/Perm kit (Becton Dickinson). Analysis of fluorescence staining was performed with a FACScan flow cytometer using CELLQuest Software (Becton Dickinson).

### Flow cytometry for cell cycle analysis

Cells were harvested and fixed in ethanol (70 % v/v) and PBS (30% v/v) at −20°C, then stored in 4°C for at least 2 hours. Fixed cells were rehydrated, stained with propidium iodide (40μg/ml) and RNase (10μg/ml) and analysed for DNA content (FACScan flow cytometer, BD Bioscience, Oxford, UK). 10,000 cells were counted for each sample and analysed using WinMDI 2.8 software (Joe Trotter, Scripps Institute, free download).

### Caspase –Glo 3/7 assays

Caspase 3/7 activity was determined using a Caspase-Glo ® 3/7 Assay Kit (Promega UK Ltd., Southampton, UK) as a measure of apoptosis. Cells were seeded in 96 well plates, and were tested according to the manufacturer's instructions. Following 48 hours of exposure to Nutlin-3 or MI-63, a 1:1 volume of Caspase-Glo 3/7 reagent was added to the wells for 1h, the well contents transferred to a white opaque plate and then analysed using a microplate luminometer (Berthold Technologies, Herefordshire, UK).

### p53 Sequencing and mutation specific PCR

Total DNA was extracted using a DNeasy Blood & Tissue Kit (QIAGEN Inc., UK) and PCR used to amplify exons 4-9 of TP53. PCR products were purified using a QIAquick PCR purification kit (QIAGEN Inc., UK) and sequenced by PCR based Sanger dideoxy chain termination. Mutations detected by sequencing were then investigated by mutation specific PCR. Primer pairs specific to either wild-type or mutant TP53 were designed as follows:

*codon 98*: sense wild-type 5′-GCCCCTGTCATCTT CTGTCCC-3′,

sense mutant 5′- GCCCCTGTCATCTTCTGTC CA-3′,

anti-sense 5′-ATACGGCCAGGCATTGAAGT-3′;

*codon 152*: sense wild-type 5′-GGTTGATTCCACA CCCC-3′,

sense mutant 5′-GGTTGATTCCACACCCA-3′,

anti-sense 5′-GGGCCAGACCTAAGAGCAAT-3′;

*codon 176*: sense wild-type 5′-GGAGGTTGTGA GGCGCTG-3′,

sense mutant 5′-GGAGGTTGTGAGGCGCTT-3′

anti-sense GGAGGGCCACTGACAACCA

*codon 285*: sense wild-type 5′- GGAGAGACCG GCGCACAG-3′

sense mutant 5′- GGAGAGACCGGCGCACAA-3

anti-sense 5′- TCCACTGATAAGAGGTCCC-3′

Following PCR, products were run on 2% agarose gels containing Ethidium Bromide and were visualised by UV light (Biorad imaging system).

### Fluorescence *in situ* hybridisation (FISH)

Cells were washed with PBS, fixed in 3:1 Methanol: Acetic Acid, and FISH preformed using a p53 (17p13) (spectrum red)/Chromosome 17 centromeric (spectrum green) probe (Kreatech Diagnostics, Amsterdam, Holland) according to the manufacturer's instructions. Results were analysed by fluorescence microscopy with two independent assessors, and a total of 100 interphase cells were scored. The percentage of cells with each differing number of red and green signals was determined and a 5% cut off applied to remove false positives. Cells containing either 1 red and 2 green signals, or 0 red and 2 green signals, were considered to have lost either one or both p53 alleles respectively.

### Immunofluorescence detection of γ-H2AX staining

Immunofluorescence detection of γ-H2AX was carried out as described in [40] apart from changes to the drug treatment schedule.

## SUPPLEMENTARY FIGURES


