# Supplementary material for: TP53 mutant MDM2-amplified cell lines selected for resistance to MDM2-p53 binding antagonists retain sensitivity to ionizing radiation
Source: Oncotarget. 2016 Jun 15;7(29):46203–18. doi: 10.18632/oncotarget.10073 (PMC5216791; doi:10.18632/oncotarget.10073)
Supplement: Supplementary file 1 [file oncotarget-07-46203-s001.pdf]

TP53 mutant *MDM2*-amplified cell lines selected for resistance to MDM2-p53 binding antagonists retain sensitivity to ionizing radiation

SUPPLEMENTARY FIGURES

A

Powerplex16 Loci	ATCC reference CRL-2098	Customer sample SJSA-1
AMELO	X,Y	X,Y
D3		16,17
THO1	7,9	7,9
D21		28,33.2
D18		15,17
PentaE		11,14
D5	12,12	12,12
D13	12,12	12,13*
D7	9,11	9,11
D16	9,10	9,10
CSF	10,11	10*,11
PentaD		2.2,10
VWA	19,19	19,19
D8		13,15
TPOX	8,9	8,9
FGA		22,23

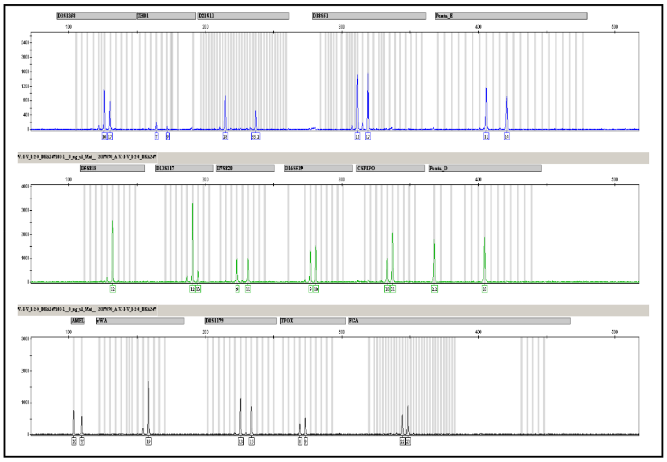

Powerplex16 Loci	ATCC reference CRL-2098	Customer sample SM6R1
AMELO	X,Y	X,Y
D3		16,17
THO1	7,9	7,9*
D21		28,33.2
D18		15,17
PentaE		11,14
D5	12,12	12,12
D13	12,12	12,12
D7	9,11	9,11
D16	9,10	9,10
CSF	10,11	10,11
PentaD		2.2,10
VWA	19,19	19,19
D8		13,15
TPOX	8,9	8,9
FGA		22,23

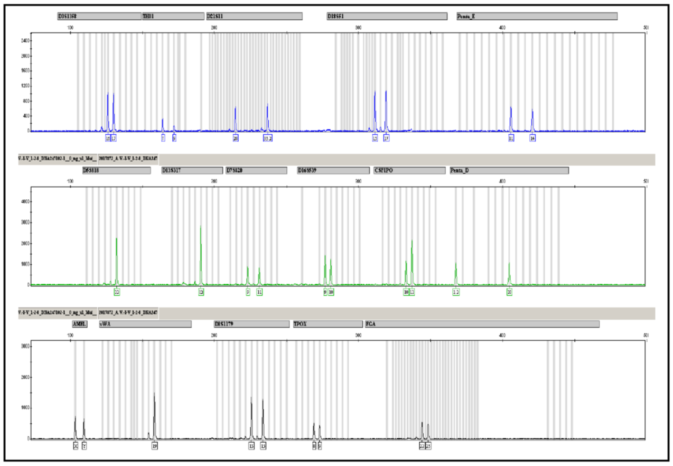

Powerplex16 Loci	ATCC reference CRL-2098	Customer sample SN40R2
AMELO	X,Y	X,Y
D3		16,17
THO1	7,9	7,9
D21		28,33.2
D18		15,17
PentaE		11,14
D5	12,12	12,12
D13	12,12	12,12
D7	9,11	9,11
D16	9,10	9,10
CSF	10,11	10,11
PentaD		2.2,10
VWA	19,19	19,19
D8		13,15
TPOX	8,9	8,9
FGA		22,23

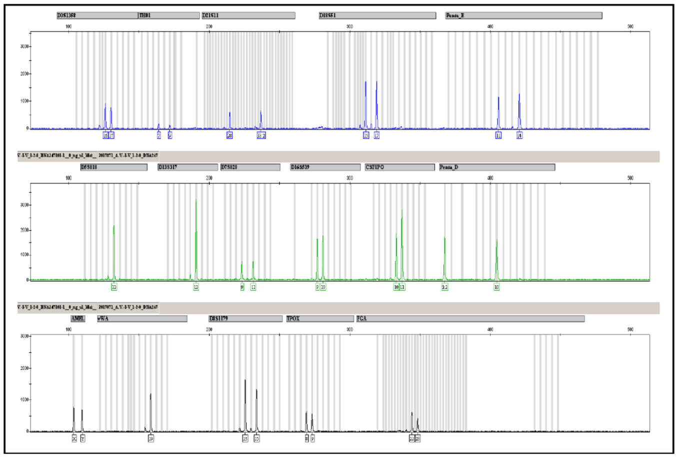

(*Continued*)

B

Powerplex16 Loci	DSMZ reference ACC 676	Customer sample NGP NM5R1
AMELO	X,Y	X,Y
D3		17,17
THO1	9,9	9,9.3
D21		32,33.1
D18		15,16
PentaE		11,15*
D5	11,12	11,12
D13	8,12	8,12
D7	11,11	11,11
D16	8,11	8,11
CSF	11,12	11,12
PentaD		8,10
VWA	16,21	16,21
D8		11,12
TPOX	6,9	6,9
FGA		19,23

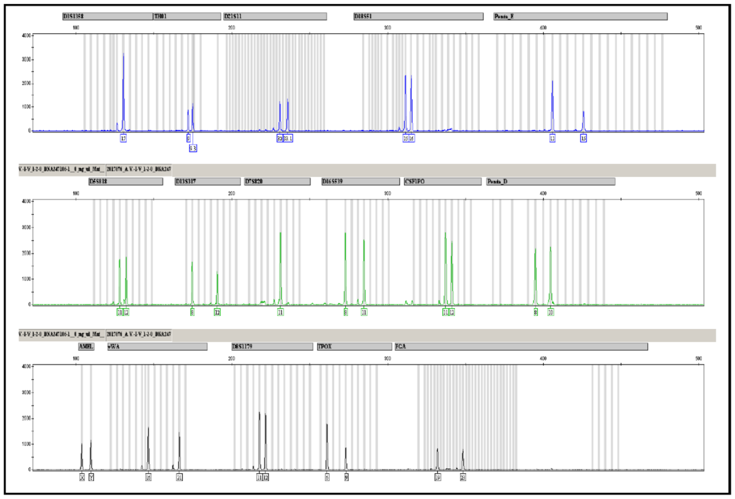

Powerplex16 Loci	DSMZ reference ACC 676	Customer sample NGP N20R1
AMELO	X,Y	X,Y
D3		17,17
THO1	9,9	9,9.3
D21		32,33.1
D18		15,16
PentaE		11,15
D5	11,12	11,12
D13	8,12	8,12
D7	11,11	11,11
D16	8,11	8,11
CSF	11,12	11,12
PentaD		8,10
VWA	16,21	16,21
D8		11,12
TPOX	6,9	6,9
FGA		19,23

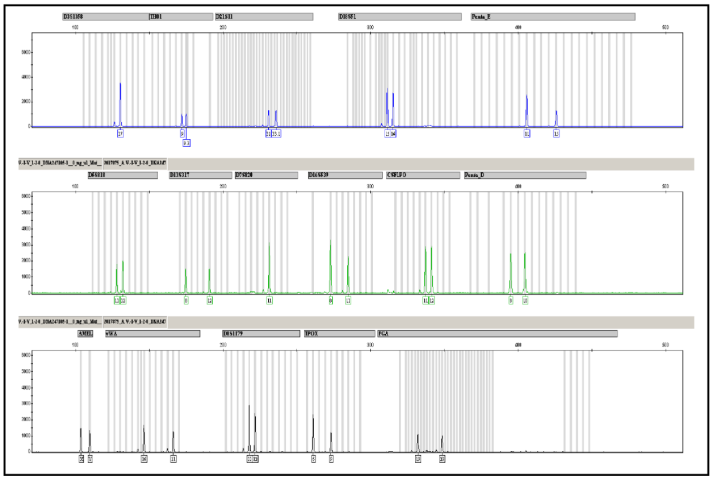

Supplementary Figure S1 (*Continued*): Cell line authentication by Short tandem repeat (STR) profiling shows that the resistant cell lines were derived from their parental cell lines. S_N40R2 and S_M6R1 cells had the same STR profile as the ATCC reference profile for SJSA-1 cells (CRL-2098) (S1A). N_NM5R2 (a different clone) and N_N20R1 had the same STR profile as the ATCC reference profile for NGP cells (ACC 676) (S1B).

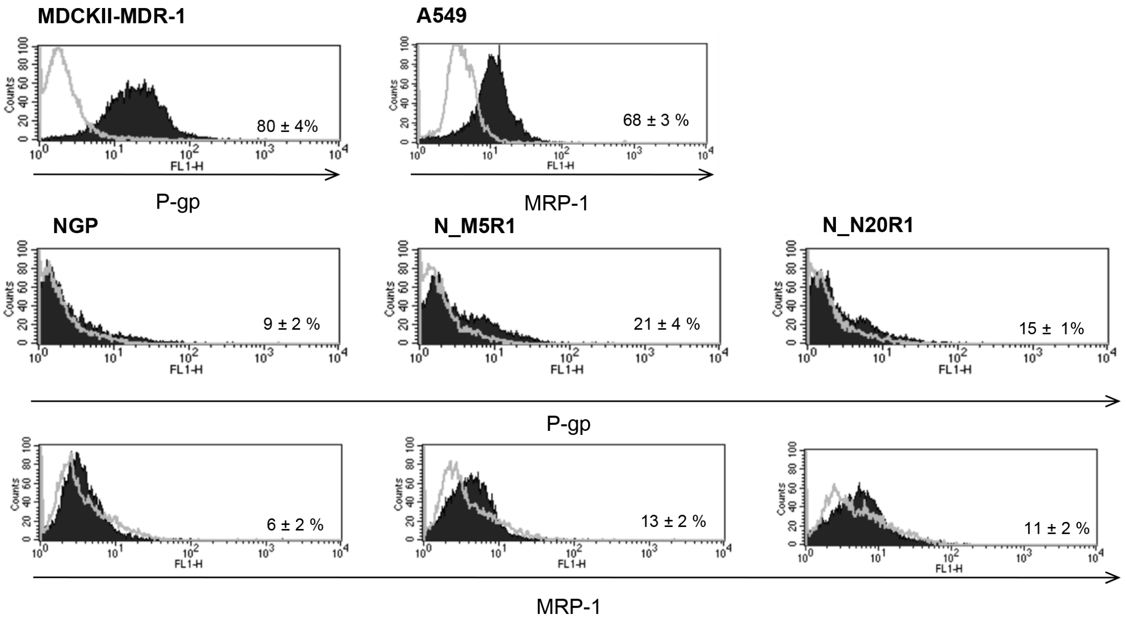

Supplementary Figure S2: P-gp and MRP-1 expression determined by flow cytometry. Results are depicted as histograms, with an increase in fluorescence (X axis, log scale) indicative of increased protein expression.

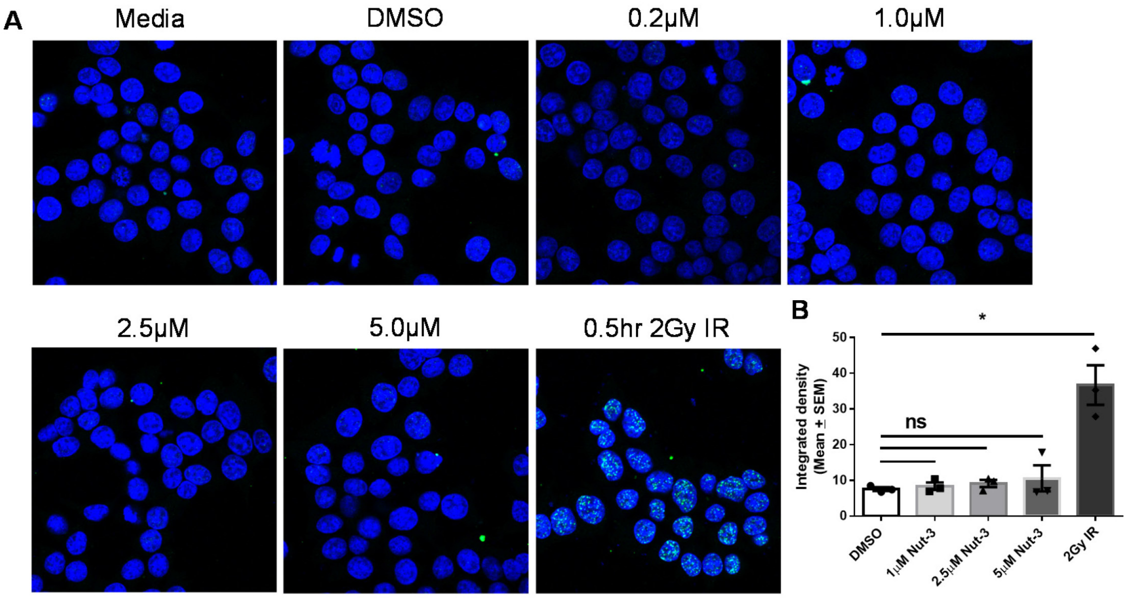

Supplementary Figure S3: γ-H2AX staining following Nutlin-3 treatment. γ-H2AX immunofluorescent staining in MCF-7 cells 30min following exposure to the stated doses of Nutlin-3. γ-H2AX signal, 30min after exposure to 2Gy IR was used as a positive control **A.** Integrated density of γ-H2AX fluorescent signals per nuclei was measured for at least 100 cells/slide using ImageJ Software for each biological repeat **B.**

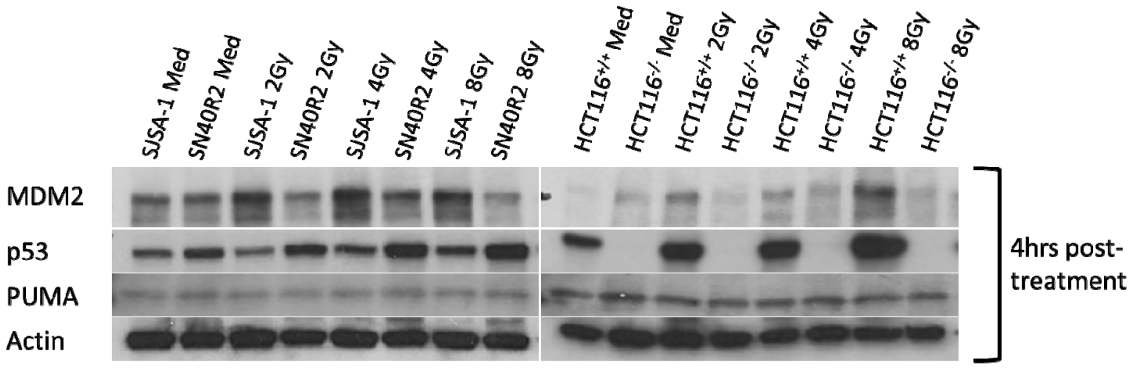

Supplementary Figure S4: p53, MDM2 and PUMA protein expression. Determined by western blotting 4 hours following exposure to ionizing radiation in parental and resistant SJSA-1 cell lines as well as HCT116 p53 +/+ and -/- cells.

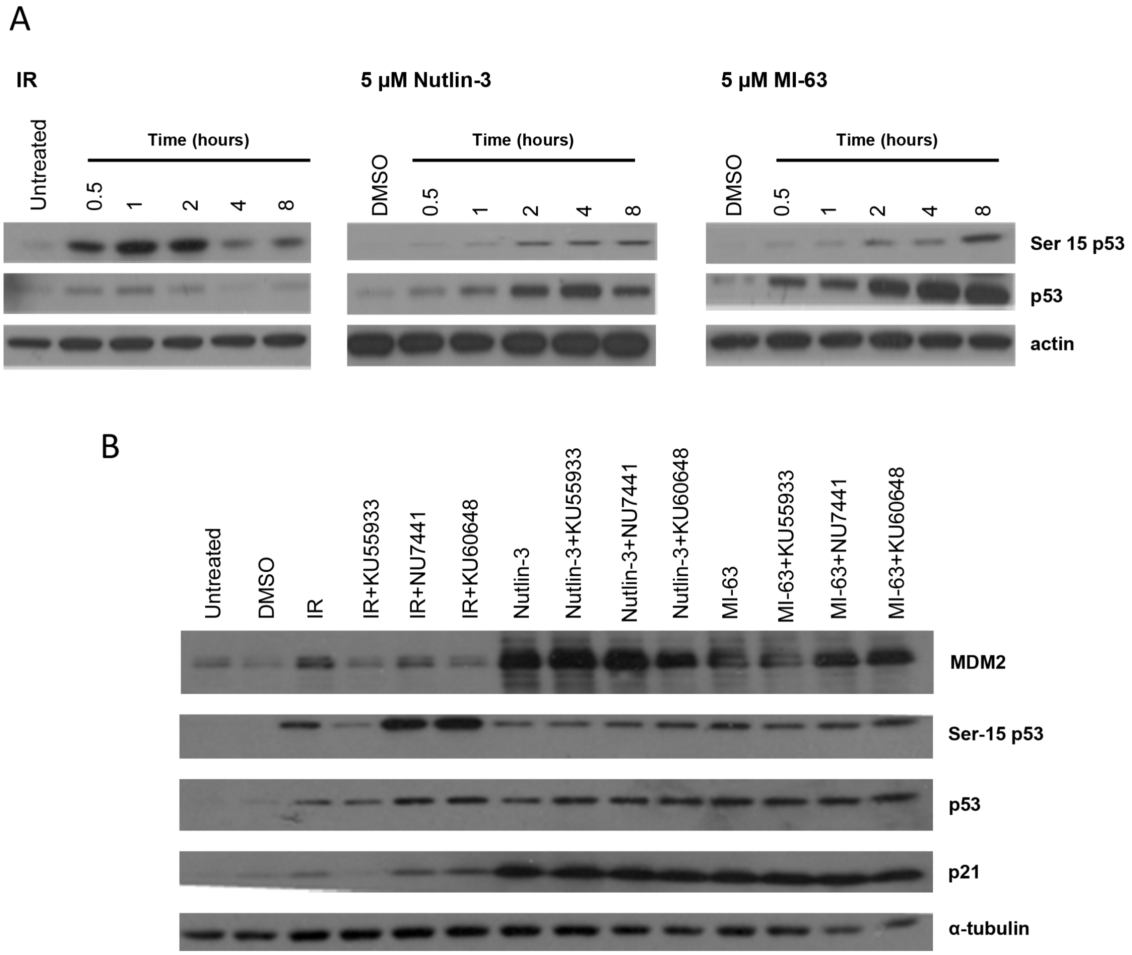

Supplementary Figure S5: Phosphorylation of p53 ser-15 in response to MDM2-p53 binding antagonists in response to non-genotoxic activation of p53. Time course of p53 ser-15 phosphorylation and increases in total p53 protein determined in parental SJSA-1 cells following exposure to either IR (6.3 Gy X-rays) or in response to continuous exposure to either nutlin-3 or MI-63 **A.** Increases in total p53, ser-15 p53, MDM2 and $p21^{WAF1}$ protein expression was determined in SJSA-1 cells exposed to either IR, nutlin-3 or MI-63 alone, or in combination with ATM and DNA-PK specific inhibitors KU55993 (10 µM), NU7441 (1 µM) and KU 60648 (1 µM) **B.**
